# Is poor oral health a risk factor for idiopathic granulomatous mastitis?

**DOI:** 10.17305/bb.2024.10324

**Published:** 2024-10-01

**Authors:** Semih Sağlık, Enver Ay, Şilan Bilek Olgaç, Necip Nas, Bilal Altunışık

**Affiliations:** 1 Department of Radiology, Faculty of Medicine, Siirt University, Siirt, Turkey; 2 Department of General Surgery, Siirt Training and Research Hospital, Siirt, Turkey; 3 Siirt Oral and Dental Health Center, Siirt, Turkey; 4 Department of Internal Medicine, Siirt Training and Research Hospital, Siirt, Turkey

**Keywords:** Idiopathic granulomatous mastitis (IGM); oral health; dental health; Decayed, Missing and Filled Teeth (DMFT) index; Simplified Oral Hygiene Index (OHI-S)

## Abstract

Idiopathic granulomatous mastitis (IGM) is a rare inflammatory breast disease that can be clinically and radiologically mistaken for carcinoma. Although its etiology remains uncertain, potential associations with pregnancy, lactation, hormonal imbalances, autoimmunity, smoking, and various microorganisms have been suggested. This study aimed to evaluate the relationship between IGM and oral health. We included 42 female patients diagnosed with IGM based on histopathological evaluations conducted between September 2018 and October 2023. The reference group consisted of 47 female patients with clinically, radiologically, and laboratory-proven nonspecific mastitis and 36 healthy female individuals. The oral health of all participants was evaluated by an experienced dentist using the “Decayed, Missing and Filled Teeth” (DMFT) index and the “Simplified Oral Hygiene Index” (OHI-S). The ages of IGM patients included in this study ranged from 29 to 51 years, with a mean age of 34.88 ± 4.87 years. The most common clinical findings were pain (*n* ═ 38), palpable breast mass, erythema, induration, and dermal sinus. Comparison of the OHI-S and DMFT index values among participants revealed that those diagnosed with IGM had significantly higher values than those in the reference group (*P* < 0.05). Our findings suggest a potential involvement of poor oral health in the etiology of IGM. Future studies should consider oral health as a factor in IGM etiology and explore the oral microbiota (OMB) in samples obtained from the affected tissue.

## Introduction

Idiopathic granulomatous mastitis (IGM) is a rare inflammatory breast disease that can be clinically and radiologically mistaken for carcinoma [1]. It was first described by Kessler and Wolloch in 1972 [2]. Clinically, it most commonly presents with breast pain, skin erythema, palpable mass, nipple retraction, edema, ulceration, and fistula [3]. It frequently shows unilateral involvement and has a recurrence rate of 16%–50%. Since it clinically and radiologically mimics malignant lesions, a histopathological examination is necessary for a differential diagnosis [4]. Although the etiology of IGM remains uncertain, it is suggested that it may be associated with pregnancy, lactation, hormonal imbalances, autoimmunity, smoking, α1-antitrypsin deficiency, and various microorganisms [3, 5]. The definitive diagnosis of IGM is achievable by excluding histopathological features of malignant lesions as well as secondary causes that lead to granulomatous inflammation, such as sarcoidosis, granulomatous polyangiitis, tuberculosis, and fungal infection [5]. On histopathological examination, the diagnosis is confirmed through the detection of non-caseating multiple granulomas with an inflammatory reaction disrupting the breast lobules [6].

Poor oral health is recognized as a significant public health problem worldwide. Numerous epidemiological studies have shown that alterations in the oral microbiome not only affect the presence and severity of oral lesions but are also associated with various systemic diseases [7–16]. The oral microbiota (OMB) may induce systemic diseases through various mechanisms. Among the most accepted mechanisms are the spread of oral infection outside the oral cavity, the entry of microbial toxins into the systemic circulation, and systemic inflammation caused by immunological reactions against soluble pathogen antigens [17, 18].

This study aims to evaluate the relationship between IGM and oral health.

## Materials and methods

This single-center study was designed as a comparative, cross-sectional, prospective study at our tertiary academic medical center. The sample size for this study was determined using G*Power software (version 3.1.9.7). The power was set at 80%, and the alpha and effect size were maintained at 0.05 and 0.3, respectively. Based on the results of the analysis, the minimum required sample size was calculated to be 111. However, to account for potential data loss, 125 patients were ultimately included in the study.

Our study involved 42 female patients who underwent ultrasonographic (USG) evaluation between September 2018 and October 2023. They were diagnosed with IGM following clinical, laboratory, radiological, and histopathological evaluations, which included a USG-guided tru-cut breast biopsy for each patient. Hematoxylin and eosin (H&E) staining for histopathological examination, Gram staining for the detection of microorganisms, Ziehl–Neelsen (ZN) staining for the identification of tuberculosis, and Periodic acid-Schiff (PAS) staining for the detection of fungal infections were conducted on all pathological samples. These procedures aimed to rule out secondary causes of granulomatous mastitis. Furthermore, serum angiotensin-converting enzyme (ACE) levels and chest X-rays were analyzed for sarcoidosis, while the cytoplasmic anti-neutrophil cytoplasmic antibodies directed against proteinase 3 (C-ANCA PR-3) levels and the spot urine protein/creatinine ratio were assessed by ELISA and immunofluorescence for granulomatous polyangiitis. Patients with a history of trauma or the presence of a foreign body were excluded from the study. The breast biopsy tissue samples of these patients revealed chronic active inflammation characterized by the presence of inflammatory cells, including polymorphonuclear leukocytes, lymphocytes, and plasma cells, as well as granuloma structures composed of epithelioid histiocytes and multinucleated giant cells. The control group included 47 female patients with clinically, radiologically and laboratory-confirmed non-specific mastitis, and 36 healthy females. Exclusion criteria encompassed patients with systemic diseases, those who had received periodontal treatment within the last six months, had a history or suspicion of known malignancy, diabetes mellitus, previous tuberculosis or contact history, and those recently vaccinated against tetanus.

Oral examinations were performed by an experienced dentist once informed consent had been obtained from all participants in the study. To evaluate oral health, our study utilized the “Decayed, Missing, and Filled Teeth” (DMFT) and “Simplified Oral Hygiene Index” (OHI-S) indices. The DMFT index quantifies the prevalence of caries by tallying the number of decayed, missing, and filled teeth [19]. The evaluation of each tooth’s condition for the DMFT index involved both clinical examination and radiographic assessment, adding together the counts of untreated caries (decayed [D]), missing teeth (missing [M]), and filled teeth (filled [F]). In instances where a tooth exhibited both caries and a filling, it was counted only once.

The OHI-S index is an evaluative tool designed to reflect an individual’s oral hygiene status through the assessment of plaque and calculus. This index system involves the examination of three specific regions, namely, the right-posterior region, left-posterior region, and anterior region of both the lower and upper jaws [20]. A total of 12 separate measurements for both plaque and calculus were performed on 6 designated teeth. In our study, buccal surfaces of upper first molars, lingual surfaces of the lower first molars, and labial surfaces of the upper right and lower left incisors were evaluated. The plaque and calculus indices were calculated separately, and their combined total was used to determine the overall oral hygiene index.

### Ethical statement

This study was conducted in adherence to the principles outlined in the Declaration of Helsinki. Approval was obtained from the Siirt University Non-Invasive Ethics Committee (Decision No: 86081). Written informed consent was obtained from all participants in this study.

### Statistical analysis

The SPSS 20.0 software (Statistical Package for the Social Sciences, Chicago, IL, USA) is used for data analysis. Data pertaining to qualitative variables are presented as number (*n*) and percentage (%), while quantitative variables are expressed as mean ± standard deviation (SD).

In analyzing the study data, the Shapiro–Wilk test was employed to determine whether the continuous variables followed a normal distribution. The Student’s *t*-test was used for comparisons between two independent groups, and one-way analysis of variance (ANOVA) was applied for comparing more than two groups, with the Tukey test conducted for post-hoc intergroup differences. Depending on the sample sizes, either the chi-square test or Fisher’s exact test was performed for categorical variables. The Pearson correlation coefficient was used to analyze the relationship between all quantitative variables. A significance level of *P* < 0.05 was accepted for statistical results.

## Results

**Figure 1.**
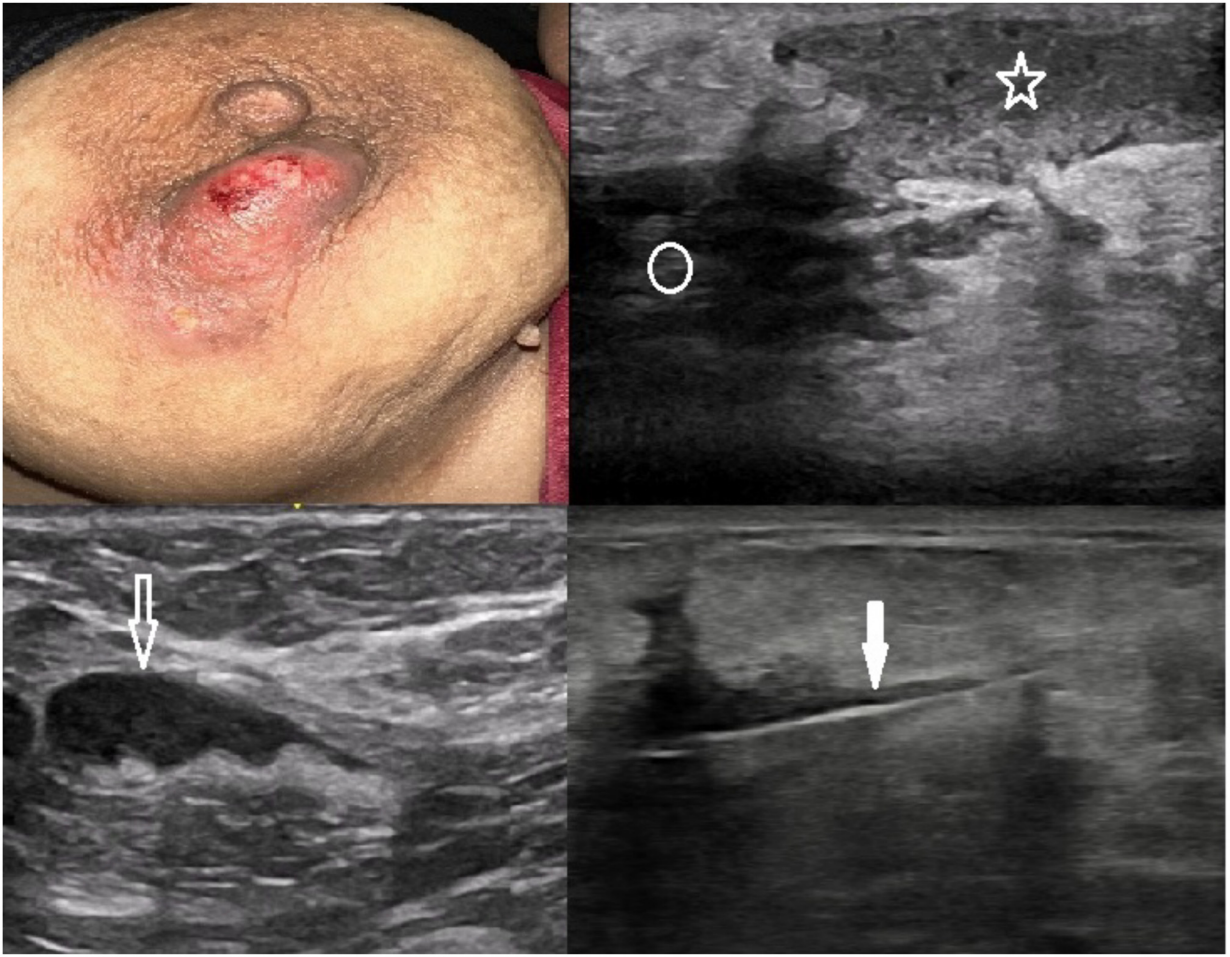
**Clinical and ultrasonographic (USG) findings in a 34-year-old woman diagnosed with idiopathic granulomatous mastitis.** The patient presented with pain, swelling, and erythema in the areolar region of the left breast. USG examination revealed an irregular hypoechoic lesion (indicated by a circle) within the breast tissue, a collection area (marked with a star) consistent with a superficial abscess beneath the skin, and a lymph node demonstrating asymmetric cortical thickening (denoted by an open arrow) in the axilla. Additionally, the image shows a USG-guided tru-cut biopsy being performed on an irregular hypoechoic lesion (indicated by a closed arrow) in the same patient.

**Figure 2.**
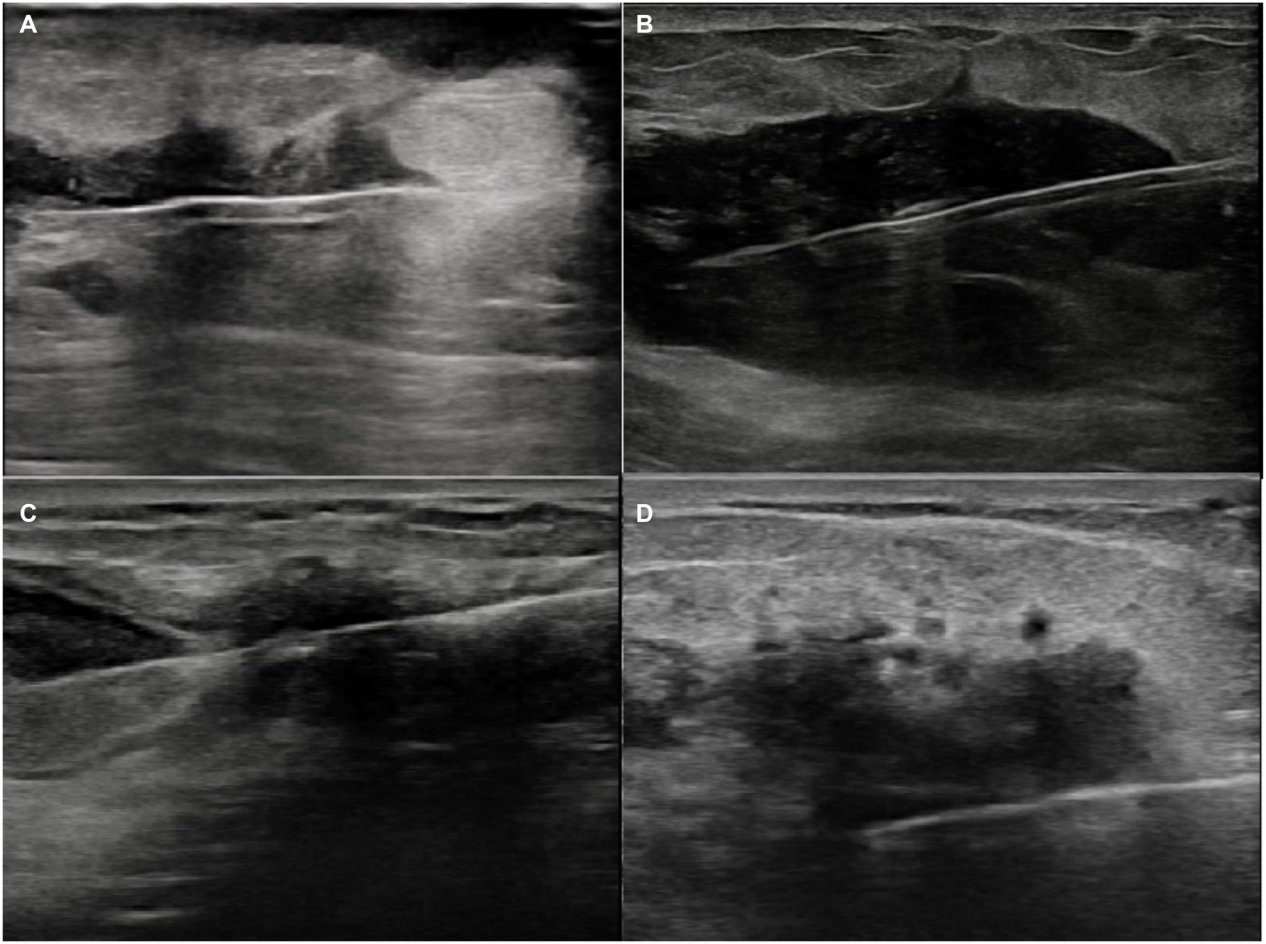
**USG-guided tru-cut biopsy images.** (A–D) Depicting USG-guided tru-cut biopsies of lesions within the breast parenchyma from four different patients diagnosed with idiopathic granulomatous mastitis. USG: Ultrasonography.

The ages of the IGM patients included in this study ranged between 29–51 years, with a mean age of 34.88 ± 4.87. The most common clinical findings were pain (*n* ═ 38), palpable breast mass (*n* ═ 32), erythema (*n* ═ 22), induration (*n* ═ 14), and dermal sinus (*n* ═ 10) (Figure 1). While bilateral breast involvement was not observed in any patient, 25 patients exhibited right breast involvement and 17 patients had left breast involvement. USG was performed on all patients, and mammography was performed on two patients. All IGM patients underwent USG-guided tru-cut biopsy, which served both for diagnosis and exclusion of malignancy, with the diagnosis subsequently confirmed through histopathology (Figure 2). The clinical and USG findings of the patients are shown in Table 1.

**Table 1:** Clinical and ultrasonographic findings in patients with idiopathic granulomatous mastitis

	***N***	**%**
*Clinical findings*		
Mass	32	76.2
Pain	38	90.4
Erythema	22	52.4
Induration	14	33.3
Sinus or ulcer	10	23.8
*Location*		
Right breast	25	59.5
Left breast	17	40.5
Bilateral breast	0	0
*Imaging findings*		
Ill-defined, irregular, heterogeneous lesions	12	28.5
Multiloculated abscess collections	10	23.8
Well-circumscribed hypoechoic mass	8	19.1
Well-circumscribed hypoechoic mass and heterogeneous lesions	5	11.9
Multiloculated abscess collections and heterogeneous lesions	7	16.6
Axillary lymphadenopathy	6	14.2

The cases included in this study were categorized into three groups. Group 1 included healthy female subjects with no known systemic or breast diseases. Group 2 comprised female patients who were clinically, radiologically, and laboratory-diagnosed with nonspecific mastitis. Group 3 included female patients with a histopathological diagnosis of IGM. Information regarding the smoking history, menopausal status, history of lactation and hormonal contraceptive use, pregnancy history, body mass index (BMI) values, and external brushing habits of the individuals in all three groups is detailed in Table 2.

**Table 2:** Demographic and clinical characteristics among healthy control individuals (group 1), nonspecific mastitis patients (group 2), and idiopathic granulomatous mastitis patients (group 3)

**Characteristics**	**Group 1 (*n* ═ 36)**	**Group 2 (*n* ═ 47)**	**Group 3 (*n* ═ 42)**	***P***
Age, mean (SD), years	32.83 ± 6.83	32.68 ± 6.76	34.88 ± 4.87	0.196
BMI, mean (SD), kg/m^2^	26.16 ± 4.61	25.4 ± 4.65	25.38 ± 3.76	0.669
Brushing teeth, *n* (%)				0.578
Regular*	17 (42.2%)	27 (57.4%)	22 (52.4%)	
Irregular**	19 (52.8%)	20 (42.6%)	20 (47.6%)	
Menopausal status, *n* (%)				0.794
Premenopausal	32 (88.9%)	42 (89.4%)	38 (90.4%)	
Perimenopausal	3 (8.4%)	3 (6.4%)	3 (7.1%)	
Postmenopausal	1 (2.7%)	2 (4.2%)	1 (2.5%)	
Smoking, *n* (%)				0.546
Yes	4 (11.1%)	5 (10.6%)	5 (11.9%)	
No	32 (88.9%)	42 (89.4%)	37 (88.1%)	
Oral contraceptives, *n* (%)				0.486
Yes	10 (27.8%)	11 (23.4%)	12 (28.5%)	
No	26 (72.2%)	36 (76.6%)	30 (71.5%)	
Breast feeding, *n* (%)				0.656
Yes	29 (80.5%)	40 (85.1%)	37 (88.1%)	
No	7 (19.5%)	7 (14.9%)	5 (11.9%)	
Gestation, *n* (%)				0.596
Yes	32 (88.9%)	44 (93.6%)	40 (95.2%)	
No	4 (11.1%)	3 (6.4%)	2 (4.8%)	

*P* < 0.05 was considered statistically significant. *Indicates individuals with the habit of brushing their teeth at least once a day. **Indicates individuals without the habit of brushing their teeth regularly (never or only occasionally). BMI: Body mass index; SD: Standard deviation.

**Figure 3.**
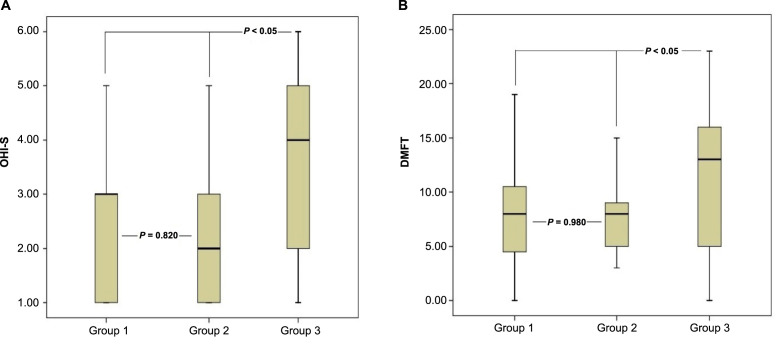
**Box plots illustrating the comparative analysis of OHI-S (A) and DMFT (B) index values across groups.** The horizontal line within each box depicts the median value, while the lower and upper rows of each box represent the minimum and maximum values, respectively. Group 1 included healthy control individuals. Group 2 comprised female patients who were diagnosed with nonspecific mastitis. Group 3 included female patients with a histopathological diagnosis of IGM. OHI-S: Simplified Oral Hygiene Index; DMFT: Decayed, Missing and Filled Teeth; IGM: Idiopathic granulomatous mastitis.

Upon comparison of the OHI-S and DMFT index values among the groups, individuals diagnosed with IGM had OHI-S and DMFT index values that were statistically significantly higher than those of individuals in both group 1 and group 2 (*P* < 0.05). However, no statistically significant difference was observed between group 2 and group 3 concerning both OHI-S and DMFT index values (*P* ═ 0.820 and *P* ═ 0.980, respectively) (Table 3 and Figure 3).

**Table 3:** OHI-S and DMFT index values among healthy control individuals (group 1), nonspecific mastitis patients (group 2), and idiopathic granulomatous mastitis patients (group 3)

	**Group 1 (*n* ═ 36)**	**Group 2 (*n* ═ 47)**	**Group 3 (*n* ═ 42)**	***P***
OHI-S, mean ± SD	2.5 ± 1.2	2.3 ± 1.6	3.4 ± 1.6	<0.05^a^
Group 1 vs Group 2				0.820^b^
Group 1 vs Group 3				<0.05^b^
Group 2 vs Group 3				<0.05^b^
DMFT, mean ± SD	8.1 ± 4.4	7.8 ± 4.2	11.3 ± 6.2	< 0.05^a^
Group 1 vs Group 2				0.980^b^
Group 1 vs Group 3				< 0.05^b^
Group 2 vs Group 3				< 0.05^b^

^a^*P* < 0.05 was considered statistically significant based on a one-way ANOVA. ^b^*P* < 0.05 was considered statistically significant based on a one-way ANOVA with a post hoc Tukey test. OHI-S: Simplified Oral Hygiene Index; DMFT: Decayed, Missing and Filled Teeth; SD: Standard deviation; ANOVA: Analysis of variance.

## Discussion

This study was conducted to investigate whether OHI-S and DMFT index values, which are oral health indicators, are associated with IGM. The main finding was that these indices were statistically significantly higher in patients with IGM compared to those in the reference groups. Variables, such as age, BMI, menopausal status, breastfeeding history, pregnancy, use of oral contraceptives (OCS), smoking history, and tooth brushing habits did not show significant differences between the three groups.

While IGM is predominantly observed in younger middle-aged individuals (3rd and 4th decades), literature reports have indicated its presence across a wider age spectrum, ranging from 11 to 83 years [3]. In our study, the mean age of the IGM cases was 34 years, aligning with the published data. Although IGM is typically present in young women with a history of lactation, there have been rare reports of male patients in the literature [21].

IGM may present clinically with a variety of symptoms and clinical findings, including a painful or painless palpable mass, skin redness, tenderness, sinus formation, ulceration, nipple discharge, and abscess formation [22, 23]. The most commonly reported clinical finding is a palpable painful mass [4, 22]. Consistent with this, the most frequent clinical finding among the IGM patients in our study was a palpable painful breast mass. Fever is generally not an expected symptom [24]. Correspondingly, none of the patients diagnosed with IGM in our study exhibited fever as a symptom or clinical finding. IGM typically presents with unilateral breast involvement, with a predominance for the right breast, while bilateral involvement is rare [1, 4–6]. In line with the literature, all IGM cases in our study displayed unilateral involvement with a predominance of the right breast.

Various factors, including autoimmunity, OCS use, infectious agents, hormonal imbalances, pregnancy, hyperprolactinemia, and α1-antitrypsin deficiency, have been accused in the etiology of IGM, though none have been conclusively proven [1, 3, 5, 22, 23]. Given that IGM typically affects women of reproductive age with a history of pregnancy and lactation, it has been thought that these factors may be the main underlying cause in the etiology [5, 25, 26]. It is thought that during lactation, the extravasation of secretions may damage the epithelium and trigger a granulomatous inflammatory response [27]. However, the occurrence of IGM in male patients reported in the literature, as well as in a wide age range of 11–83 years and in patients without a history of lactation, suggests that lactation and pregnancy alone cannot be solely responsible for its development [3, 28, 29]. Furthermore, the fact that five patients (11.9%) in our study had no history of lactation and one patient (2.4%) was postmenopausal indicates the influence of other risk factors in the etiology of IGM.

The use of OCS has also been suggested as a contributing factor in the etiology of IGM, postulated to be a risk factor due to its potential to increase breast secretion. Binesh et al. [30] reported that the frequency of OCS use in IGM was 36%. Aghajanzadeh et al. [31] noted a higher susceptibility to IGM among OCS users. However, Altintoprak et al. [3] reported a variable association between IGM and OCS use, ranging from 0% to 42%, concluding that there is no significant relationship between OCS use and IGM. In our study, the history of OCS use was noted in only 28.5% (12 patients) of the IGM cases, suggesting a diminished role of this factor in the etiology.

Smoking has been another factor implicated in the etiology of the disease. However, due to the widely varying associations reported in the literature, ranging from 0% to 77%, it remains uncertain whether smoking is a definitive etiological factor [32, 33]. Asoglu et al. reported that 77% of patients with IGM had a history of smoking, whereas Baslaim et al. reported no smoking history in their IGM patients [32, 33]. Additionally, Prasad et al. noted that in a study with 73 patients, only two patients (2.74%) were reported to have a history of smoking [34]. In our study, only five patients (11.9%) reported a history of smoking, which challenges the likelihood of smoking being a significant etiological risk factor.

Autoimmunity has emerged as the most widely accepted etiological theory for IGM, largely due to the positive response to steroids and immunosuppressive therapies observed in the treatment of the disease. In addition, immunohistochemical demonstration of T-lymphocyte predominance in biopsy specimens, reported in literature studies, supports this view [29, 35, 36]. It has been reported that an autoimmune response to fat or protein-rich secretion extravasated from breast lobules may cause chronic inflammation [37]. In particular, T cell-mediated inflammation is believed to be responsible for the formation of non-caseating granulomas [35]. However, a definitive etiological trigger for this autoimmune mechanism has yet to be identified [38]. The literature has discussed the co-occurrence of autoimmune diseases, such as erythema nodosum, rheumatoid arthritis (RA), multiple sclerosis (MS), systemic lupus erythematosus (SLE), and psoriasis with IGM, although these cases represent only a small fraction of the total [39–41]. In our study, one patient had psoriasis and two patients had RA, which accounted for only 7.1% of all patients.

Some microbiological agents have been implicated in the etiology of IGM. Histopathological isolation of certain bacterial species, notably *Corynebacterium*, *Streptococci*, and *Propionibacterium*, has been reported. The most frequently isolated species is *Corynebacterium* species [42–45]. In a study conducted by Taylor et al. on 62 patients, *Corynebacterium* species were isolated in 55% of the cases, with fever and fistula formation being more frequently observed in these cases [46]. However, since *Corynebacterium* species are members of the normal skin flora, it is challenging to determine whether they are causative of infection or merely contaminants [47]. Furthermore, the literature suggests that molecular-based analyses have not detected microbiological agent positivity among many flora bacteria, including common infectious agents, and no growth has been observed in culture samples [3, 48]. The assumption of a bacterial role in the etiology of IGM is further weakened by the observation that IGM patients typically do not benefit from antibiotic treatments and do not experience clinical improvement. In our study, we did not identify any bacteriological agents in culture samples obtained from IGM patients.

Due to the lack of characteristic imaging features in IGM, diagnosis through radiological methods is challenging [49]. USG is often the imaging modality of choice, given the typically young age of the patient population [50]. USG may reveal hypoechoic, heterogeneous lesions with a tubular configuration, hypoechoic masses with lobulated contours, multiloculated abscess formations, fistulas extending to the skin, or axillary lymphadenopathy [29]. Doppler USG examination usually shows increased vascularity in the affected breast parenchyma [51]. Mammography is recommended to exclude microcalcifications in cases of suspected malignancy but usually does not provide specific information. Microcalcifications are generally not an expected imaging finding in patients with IGM [50, 52]. In our study, the most frequent USG findings were hypoechoic, heterogeneous, tubular lesions observed in 28.5% (12 patients) of the patients, multiloculated abscess collections in 23.8% (10 patients) of the patients, and well-circumscribed hypoechoic mass lesions in 19% (8 patients) of the patients. Axillary lymphadenopathy was detected in 14.2% (6 patients) of the patients.

Since reliance on imaging findings for diagnosis can lead to misdiagnosis and inappropriate treatment, histopathological examination is essential for a definitive diagnosis of IGM. Fine needle aspiration biopsy (FNAB) has a low sensitivity and is considered to have a limited role in IGM diagnosis [53]. There are documented instances in the literature where patients were misdiagnosed with carcinoma based on FNAB results and consequently received incorrect treatment [54–56]. As a result, a more conclusive diagnosis requires a tru-cut biopsy or comprehensive breast tissue sampling [57]. In our study, the diagnosis of all IGM patients was established through tru-cut biopsy, with subsequent follow-up and treatment tailored to this confirmation.

The oral cavity provides an ideal environment for microorganisms, owing to its suitable temperature, humidity, and nutrient abundance [58]. A dynamic interaction exists between these microorganisms and the host organism, and any disturbance in this balance can lead to a microbial imbalance known as dysbiosis [59, 60]. In cases of dysbiosis, some microbial colonies become more widespread and may cause pathogenic effects on the host organism [60]. Dysbiotic changes have been linked to local diseases, such as dental caries and periodontal disease, and may be implicated in the etiology of various systemic diseases, including cardiovascular disease, pneumonia, malignancies, diabetes, obesity, autoimmune diseases, cystic fibrosis, and cerebral or hepatic abscesses [8–16].

Poor oral hygiene facilitates the colonization of pathogenic microorganisms in periodontal tissues. Given the anatomical proximity of periodontal tissues to the bloodstream, endotoxins and/or cytokines released by these pathogenic microorganisms can directly or indirectly enter the systemic circulation, leading to bacteremia and inflammation in distant organs [17, 61, 62]. Research has demonstrated that systemic concentrations of certain pro-inflammatory cytokines are elevated in instances of periodontal inflammation, with serum levels of these biomarkers significantly decreasing following treatment [61, 63, 64]. Consequently, poor oral health affects not only the periodontal inflammatory processes but also the systemic inflammatory status.

Periodontal inflammation or chronic bacteremia predisposes to the development of a systemic immune reaction. In recent studies, *Porphyromonas gingivalis* and *Treponema denticola* have been found to trigger a systemic immune response [65, 66]. It has been demonstrated that some periodontal pathogens can exacerbate various microvascular complications, such as nephropathy, retinopathy, and neuropathy in diabetes patients, and increase cardiorenal mortality twofold [62, 67]. Some periodontal pathogens, especially *Fusobacterium nucleatum*, have been shown to cause adverse pregnancy outcomes, such as low birth weight or stillbirth [68, 69]. Cestari et al. [70] have reported a predisposition to Alzheimer’s disease in the context of periodontal inflammation, with an increase in proinflammatory cytokines observed in affected patients. Zhang et al. [71] suggested that some periodontal pathogens could initiate a systemic inflammatory response through the hematogenous pathway in mouse models, subsequently leading to systemic osteoporosis. Bernhard et al. [72] suggested that the inflammation caused by abundant periodontal pathogens in subgingival biofilm samples from breast cancer cases might indirectly contribute to the development of the disease. Sfreddo et al. [73] reported that women diagnosed with periodontitis had a two to three times higher risk of developing breast cancer compared to healthy controls. da Silva et al. [12] suggested that a strain variant of *Streptococcus constellatus*, emerging through genetic recombination within the periodontal pocket, may have the potential to form brain abscesses. Additionally, numerous studies have shown that periodontal inflammation plays an active role in triggering or exacerbating autoimmune diseases, including SLE, primary sclerosing cholangitis, RA, Sjögren’s syndrome, and autoimmune hepatitis [74–77].

To the best of our knowledge, this is the first study to examine the relationship between poor oral health and IGM. In this study, we observed that the OHI-S index values, which indicate deficiencies in oral hygiene, and DMFT index values, which reflect poor oral health, were significantly higher in patients with a histopathological diagnosis of IGM compared to those without such a diagnosis. This finding led us to draw a connection between IGM and poor oral health. Given the observed effectiveness of steroid or immunosuppressive treatments in patients with IGM, we hypothesize that the mechanism underlying this association may be initiated or exacerbated by a distant organ inflammation. This inflammation could result from endotoxins or cytokines from the OMB entering the circulatory system due to dysbiotic changes.

## Conclusion

In conclusion, our findings suggest that poor oral health may be involved in the etiology of IGM. Therefore, it would be beneficial for future research to incorporate oral health considerations into the etiological study of IGM and to investigate the OMB in samples taken from the target tissue. Furthermore, should our findings be confirmed by subsequent studies involving a larger number of patients, precautions regarding poor oral health could contribute to decreasing the incidence of IGM.

## Data Availability

Data related to this study can be obtained from the corresponding author upon reasonable request.
